# Recent Investigations on the Use of Copper Complexes in Photovoltaic Application

**DOI:** 10.3390/nano16130830

**Published:** 2026-07-06

**Authors:** Francesco Fagnani, Alessia Colombo, Dominique Roberto, Federico Turco, Claudia Dragonetti

**Affiliations:** Department of Chemistry, Università Degli Studi di Milano, UdR-INSTM, Via C. Golgi 19, I-20133 Milano, Italy; francesco.fagnani@unimi.it (F.F.); alessia.colombo@unimi.it (A.C.); dominique.roberto@unimi.it (D.R.); federico.turco@unimi.it (F.T.)

**Keywords:** copper complexes, solar cells, DSSCs, redox mediators, dyes

## Abstract

Copper complexes have recently emerged as key materials for advancing dye-sensitized solar cells (DSSCs) toward more sustainable and high-performance photovoltaic technologies. This minireview summarizes the most significant achievements reported from 2024 onwards, highlighting the multifaceted role of copper in DSSCs as sensitizers, redox mediators, and functional components in innovative device architectures. Significant progress has been achieved in all these roles; however, the most remarkable advances concern copper-based redox mediators, where fine-tuning of ligand environments, additives, and electrolyte formulations has enabled excellent efficiencies, exceeding 10%, together with outstanding long-term stability. Developments in aqueous and quasi-solid-state systems further enhance the environmental compatibility and durability of these devices. In addition, novel concepts, including retro cells and copper-based “zombie” DSSCs, demonstrate the versatility of copper chemistry in simplifying device design and enabling new applications. Overall, these findings confirm copper complexes as highly promising earth-abundant alternatives to noble-metal-based systems although further work is still required to optimize light absorption, suppress charge recombination, and improve large-scale device stability.

## 1. Introduction

Solar cells play a crucial role in the generation of clean and renewable energy directly from sunlight, contributing to the reduction in dependence on fossil fuels, the mitigation of greenhouse gas emissions, the lowering of energy costs, and the enhancement of energy independence. In this context, considerable research efforts have been devoted to the development of efficient and sustainable photovoltaic technologies. Among third-generation photovoltaic systems, dye-sensitized solar cells (DSSCs) have emerged as one of the most extensively investigated and promising alternatives to conventional silicon-based devices.

DSSCs offer several advantages, including low fabrication costs, mechanical flexibility, semi-transparency, and the possibility of tuning their optical and electronic properties through molecular design. Moreover, they exhibit excellent performance under low-light, indoor, and diffuse illumination conditions, making them particularly attractive for emerging applications such as building-integrated photovoltaics and indoor energy harvesting.

DSSCs were first introduced in 1991 by O’Regan and Grätzel [1], marking a breakthrough in the field of photovoltaics, and have since attracted steadily increasing attention within the scientific community [2,3]. Continuous advancements in materials design, device architecture, and electrolyte engineering have significantly improved their efficiency and stability, further consolidating their potential as a viable technology for next-generation solar energy conversion.

**Figure 1:**
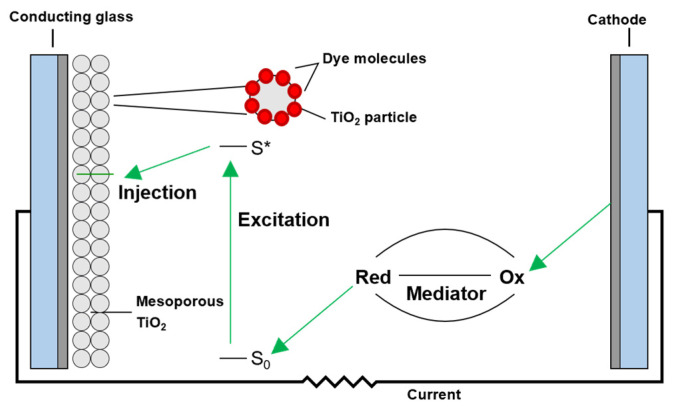
Schematic drawing a DSSC. Copper complexes can act as dye molecules on TiO_2_, or as redox mediator couple.

The operating mechanism of DSSCs is based on the sensitization of a semiconductor electrode with an organic molecule or a coordination complex acting as a photoactive dye. The sensitizer, typically containing anchoring groups such as carboxylic acid functionalities, is adsorbed onto a mesoporous TiO_2_ film deposited on a conductive glass substrate, thereby forming the photoanode. Upon absorption of photons with sufficient energy, the dye is promoted to an excited electronic state and injects an electron into the conduction band of the TiO_2_ semiconductor. The injected electron subsequently diffuses through the mesoporous network toward the transparent conducting oxide and is collected at the anode, from which it flows through an external circuit to reach the counter electrode. At the counterelectrode, the electron participates in the reduction of a redox mediator, typically the I^−^/I_3_^−^ couple, which in turn regenerates the oxidized dye at the photoanode, thereby closing the catalytic cycle. Finally, the regenerated dye returns to its ground state, enabling continuous operation of the photovoltaic process. The overall efficiency of DSSCs is governed by the delicate balance between efficient light absorption, fast electron injection, effective dye regeneration, and the minimization of charge recombination processes [1,2,3] (see Figure 1).

Among the wide variety of dyes employed as sensitizers in DSSCs, the most widely studied is the Ru-based dye N719, which is commonly used as a benchmark for the evaluation of newly developed sensitizers [4]. To date, numerous Ru(II) complexes have been investigated, initially featuring structures closely related to that of N719 and, more recently, evolving toward systems that do not contain NCS ligands, in order to improve stability and tunability [5,6,7,8].

However, ruthenium presents two major limitations, namely its scarcity and high cost. To address these issues, significant research efforts have been directed toward the development of alternative sensitizers based on earth-abundant metals. Not all first-row transition metals provide comparable performance in dye-sensitized solar cells, as ruthenium dyes. For example, iron and cobalt complexes are highly attractive from the standpoint of abundance and sustainability, but they often suffer from intrinsic limitations such as short-lived excited states, inefficient charge separation, and not optimal energy-level alignment with semiconductor electrodes and redox mediators. These drawbacks have so far hindered their widespread application in high-efficiency DSSCs. In this context, copper complexes have emerged as particularly promising candidates due to their low cost, high natural abundance, and favorable redox properties [9,10,11,12]. From a practical perspective, copper is also characterized by relatively low toxicity, making it highly suitable for the development of sustainable photovoltaic technologies. At the same time, copper exhibits unique electronic features that are especially advantageous for DSSC operation [9,10,11,12]. Another key advantage and peculiarity of copper lies in its versatile coordination chemistry. The structural flexibility of Cu(I) and Cu(II) centers allows for fine control of steric and electronic properties through rational ligand design, enabling the tuning of redox potentials, photophysical properties, and chemical stability. This tunability is particularly important in DSSCs, where a delicate balance between light absorption, electron injection, dye regeneration, and suppression of recombination processes must be achieved. In particular, the Cu(I)/Cu(II) redox couple is associated with fast outer-sphere electron-transfer kinetics and relatively low reorganization energies, which facilitate efficient dye regeneration and contribute to the achievement of high open-circuit voltages. These properties distinguish copper from many other first-row transition metals and make it especially attractive for redox mediator applications.

In fact, concerning redox mediators, the most commonly used system is the I^−^/I_3_^−^ couple, which suffers from several drawbacks. These include the volatility of I_2_ (in equilibrium with I_3_^−^), which complicates long-term device sealing; the strong visible absorption of I_3_^−^, which limits light-harvesting efficiency; and the corrosive nature of the I_3_^−^/I^−^ redox couple toward many metallic components. To overcome these limitations, extensive efforts have been devoted to the development of alternative electron shuttles. In this regard, copper-based redox couples have been successfully implemented as efficient mediators in DSSCs [12,13,14,15,16], making copper particularly attractive for photovoltaic applications. In fact, copper-based redox mediators typically exhibit weaker absorption in the visible region, improved electrochemical stability, and reduced corrosivity, which translate into higher photovoltages, improved device durability, and broader compatibility with advanced device architectures, including quasi-solid-state and aqueous systems.

Therefore, copper complexes should be regarded not merely as cost-effective substitutes for noble-metal systems, but as highly competitive and multifunctional platforms capable of overcoming several intrinsic limitations of both traditional ruthenium-based sensitizers and iodide-based electrolytes. Their unique combination of sustainability, tunability, and high performance has positioned copper at the forefront of current research in dye-sensitized solar cells and related photovoltaic technologies.

This minireview aims to summarize the most recent developments (from 2024 onwards) in the use of copper complexes in DSSCs, providing an updated perspective on the field in light of recent comprehensive reviews [3,9,10,11,12,15,16]. In particular, attention is focused on the role of copper complexes as sensitizers, redox mediators—currently the most extensively investigated application—and as functional components in emerging device concepts, as discussed in the following sections.

## 2. Results and Discussion

### 2.1. Copper Complexes as Dyes

**Figure 2:**
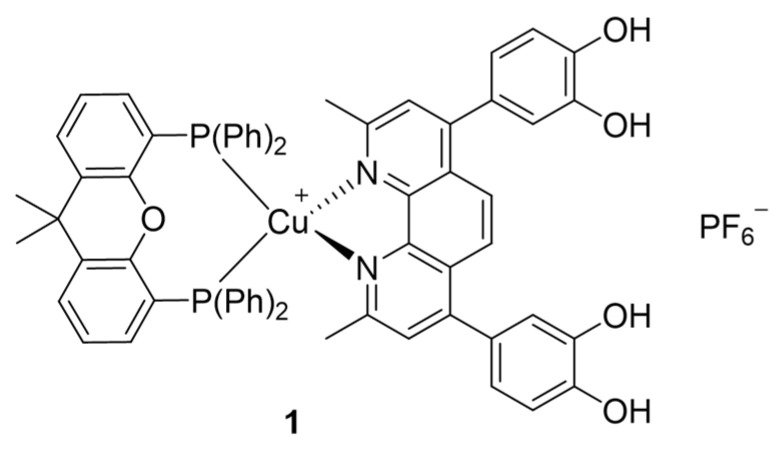
Molecular structure of the heteroleptic Cu(I) complex **1** [17].

The study by Yann Pellegrin, Michael Karnahl, and co-workers [17] reports a DSSC based on heteroleptic Cu(I) diimine–diphosphine complexes bearing catechol anchoring groups (see Figure 2), addressing two major limitations of Cu(I) sensitizers, namely weak light absorption and inefficient interfacial charge injection. The authors designed a catechol-functionalized phenanthroline ligand and incorporated it into Cu(I) complexes.

The key novelty lies in the demonstration of a dual-chromophore mechanism, in which the TiO_2_–catechol unit acts as the primary light absorber and charge injector, while the Cu(I) complex functions as an antenna, enhancing photocurrent and suppressing charge recombination. Using this approach, the heteroleptic Cu(I) dye, **1**, achieves a record power conversion efficiency (PCE) of approximately 1.9%, corresponding to a 35-fold increase compared to previously reported heteroleptic (N^N)(P^P) Cu(I)-based DSSCs [17].

A further important advance is the successful operation of these Cu(I)–catechol dyes in aqueous electrolytes, showing stable performance for at least 10 days. This result highlights catechol anchoring groups as a highly effective strategy for the development of durable, water-compatible, and noble-metal-free DSSCs.

The second study is a theoretical investigation by Conradie M.M. [18], which employs quantum chemical calculations to examine the electronic structure, charge-transfer pathways, and energetics of the molecular system under study, providing a detailed molecular-level interpretation of its photophysical and interfacial properties. By combining density functional theory (DFT) with excited-state analysis, this work elucidates how structural features influence frontier orbital localization, excitation character, and charge-separation tendencies.

The key novelty lies in the development of a theory-driven mechanistic framework that goes beyond qualitative interpretations. Specifically, the study identifies the origin of charge-transfer states and their energetic alignment with interfacial and reactive states, thereby clarifying why certain molecular designs are more favorable for efficient charge injection and stabilization. This approach provides valuable predictive design rules that can be used to rationally optimize related molecules prior to synthesis, thus offering strong support to experimental efforts.

In particular, the authors study the introduction of aromatic substituents on the β-diketonato ligands in Cu(β-diketonato)_2_ Cu(II) complexes. From a theoretical point of view, the frontier molecular orbitals are favorably aligned for efficient electron injection into the semiconductor and for dye regeneration by the iodide/triiodide redox couple.

### 2.2. Copper Complexes as Redox Mediators

Extensive research efforts have been devoted to the development of copper complexes as redox mediators in DSSCs, in combination with a wide range of organic and coordination compounds as sensitizers.

Tarek H. Ghaddar and co-workers [19] reported the development of a fully aqueous, copper-based DSSC that is environmentally friendly, low-cost, and highly stable. In this work, the authors introduce, for the first time, N-methylbenzimidazolium acetate as a multifunctional additive in an aqueous copper(I/II) redox electrolyte. This additive plays a dual role, acting both as a pH buffer and as a source of the Lewis base N-methylbenzimidazole, which effectively suppresses charge recombination and enhances dye regeneration.

**Figure 3:**
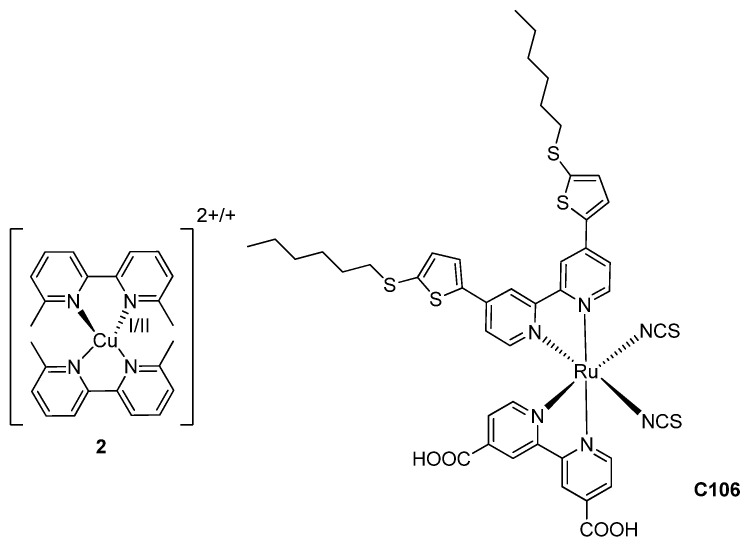
Molecular structure of the Cu(I/II) redox couple **2** and of Ru dye C106 [19].

As a result, the device exhibits excellent performance under indoor ambient lighting conditions, together with remarkable long-term durability. The optimized system, based on the Cu(I/II) complex, **2**, and the ruthenium dye C106 (see Figure 3), achieves a power conversion efficiency (PCE) of approximately 7.9% at 1000 lx. Notably, the efficiency increases to ~9.3% after one year of continuous exposure to ambient light, while the device remains operational after 1000 h at 52 °C. This outstanding level of stability and performance sets a benchmark for sustainable aqueous DSSCs and highlights their strong potential for indoor energy harvesting applications [19].

**Figure 4:**
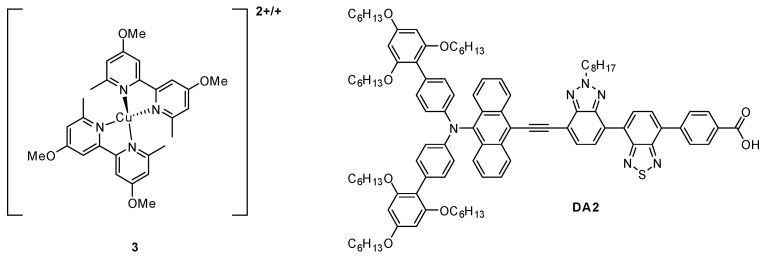
Molecular structure of the Cu(I/II) redox couple **3** and of the new dye DA2 [20].

The work of Chen-Yu Yeh and co-workers [20] presents a highly efficient and stable DSSC enabled by the rational co-design of a modified copper redox mediator and a new organic dye (DA2, see Figure 4). The authors developed a modified copper bipyridine-based redox mediator, Cu(I/II) complex, **3**, featuring methoxy-substituted ligands that suppress detrimental coordination with the additive *tert*-butylpyridine, thereby preserving fast charge transport and a high fill factor.

In parallel, the newly designed DA2 dye, characterized by a cascade acceptor architecture, enhances light harvesting and photocurrent generation. The synergistic combination of these two components results in both high performance and improved long-term stability in copper-mediated DSSCs.

The most notable achievement is the demonstration of a champion device with a power conversion efficiency of 10.2%, accompanied by excellent operational stability, retaining 88% of its initial efficiency after 95 days. This represents a significant advance for copper-based DSSCs, which have traditionally suffered from fill-factor losses and instability associated with *tert*-butylpyridine-induced degradation.

**Figure 5:**
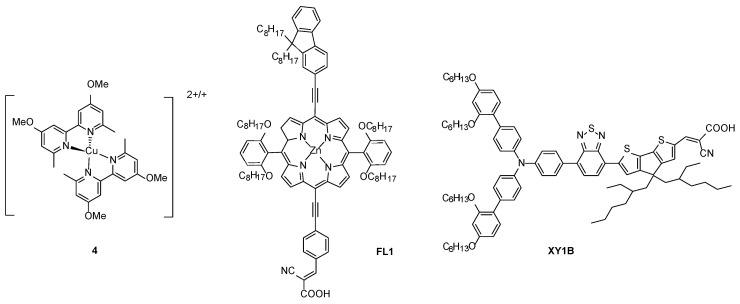
Molecular structure of the Cu(I/II) redox couple **4**, of the new dye FL1 and of the complementary organic dye XY1B [21].

The study by Hiroshi Imahori et al. [21] represents a significant advance in DSSCs, demonstrating that rational energy-level engineering of both porphyrin dyes and copper(I/II) redox shuttles can overcome the long-standing limitation of inefficient dye regeneration in copper-based DSSCs (see Figure 5). A newly designed porphyrin dye, FL1, featuring a weakly electron-donating fluorene unit and bulky alkyl chains, provides a sufficient driving force for dye regeneration while simultaneously suppressing dye aggregation and charge recombination, leading to improved photocurrent and open-circuit voltage.

When combined with an optimized copper redox shuttle based on 4,4′-dimethoxy-6,6′-dimethyl-2,2′-bipyridine ligands, **4**, the system exhibits enhanced charge transport properties, particularly when the PF_6_^−^ counterion is employed to improve diffusion within the electrolyte. Under these conditions, the DSSCs achieve a record efficiency of 9.06% for a single porphyrin dye with a copper electrolyte, much higher than that previously reported.

Importantly, co-sensitization of FL1 with a complementary organic dye (XY1B) extends the spectral response and further enhances light harvesting, resulting in a power conversion efficiency of 10.9% while maintaining a high open-circuit voltage of 0.945 V. This result represents the first porphyrin-based DSSC employing copper(I/II) redox shuttles to surpass the 10% efficiency threshold.

In addition to the efficiency gains, the devices also show improved operational stability, highlighting the potential of copper-based redox mediators in combination with carefully engineered porphyrin dyes for the development of next-generation, high-performance DSSCs.

**Figure 6:**
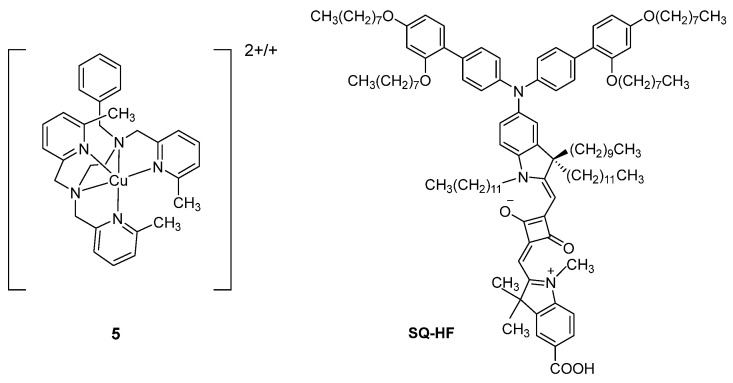
Molecular structure of Cu(I/II) redox couple **5** and of the organic dye SQ-HF [22].

Continuing with copper electrolytes and the development of new dyes, it is important to highlight the recent work of Kothandam Krishnamoorthy and Jayaraj Nithyanandhan et al. [22]. The main achievement of this study is the successful design and demonstration of a far-red/NIR-active unsymmetrical squaraine dye (SQ-HF) that is fully compatible with a copper(II/I) redox electrolyte, **5**, (see Figure 6), enabling improved DSSC performance.

By incorporating a bulky Hagfeldt donor and long alkyl chains, the authors effectively suppressed dye aggregation, reduced interfacial charge recombination, and optimized energy-level alignment for efficient electron injection and dye regeneration. These structural features are particularly important for extending absorption into the far-red and near-infrared regions while maintaining good device performance.

As a result, the SQ-HF-sensitized DSSC achieved a maximum power conversion efficiency of 5.15% (with Jsc = 10.83 mA cm^−2^ and Voc = 0.690 V) using a [Cu(tme)]^2+/+^ electrolyte (tme = N-benzyl-N,N′,N′-tris(6-methylpyridin-2-ylmethyl)ethylenediamine), **5**, in combination with a CDCA additive (CDCA = chenodeoxycholic acid).

This performance represents one of the best results reported for far-red squaraine dyes employing copper electrolytes and demonstrates, for the first time, their effective integration within this type of redox system.

**Figure 7:**
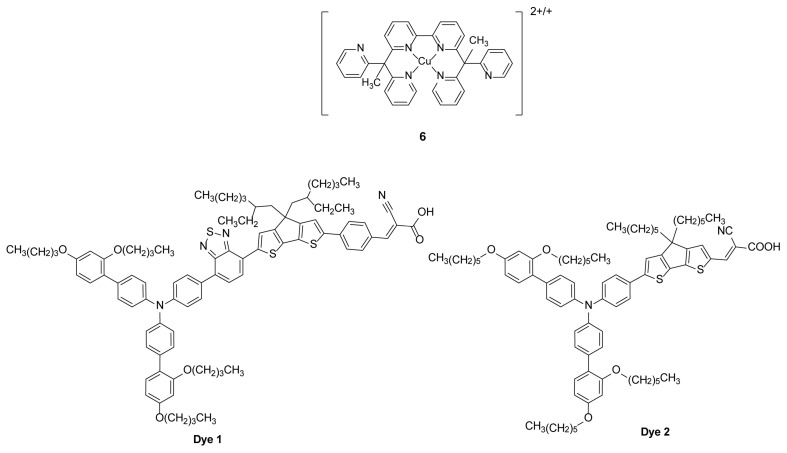
Molecular structure of complex **6** and of Dyes 1 and 2 [23].

Gupta, M.K. et al. explore copper-based redox shuttles, specifically [Cu(bpyPY4)]^+^ and [Cu(bpyPY4)]^2+^ (where bpyPY4 = 6,6-bis(1,1-di(pyridin-2-yl)ethyl)-2,2′-bipyridine), **6**, in combination with triphenylamine-based D-π-A dyes (see Figure 7). The study aims to provide detailed insights into the geometrical, optical, optoelectronic, and photovoltaic properties of these systems through density functional theory (DFT) and time-dependent DFT (TDDFT) calculations [23].

The results show that the hexadentate polypyridyl copper complex possesses a suitable redox potential (4.67 eV), good geometrical stability, and pronounced metal-to-ligand charge-transfer (MLCT) character, all of which facilitate efficient dye regeneration and help address key limitations associated with conventional iodide-based electrolytes. Comprehensive analyses of the electronic structure—including frontier molecular orbitals, density of states, molecular electrostatic potential, and optical absorption spectra—indicate that the HOMO and LUMO energy levels of the dyes are favorably aligned for efficient electron injection into TiO_2_ and rapid regeneration by the copper redox shuttle. It is important to underline that the reported data have not been experimentally proved. The indicated redox potential must be assigned to the corresponding couple and the MLCT character of the absorption bands should be attributed only to the Cu(I) species.

Moreover, key photovoltaic descriptors reveal negative electron-injection free energies, low regeneration barriers, high light-harvesting efficiencies, and the potential for enhanced open-circuit voltages. These findings further suggest that the investigated systems may also perform effectively under low-light conditions, where efficient charge transfer and minimized recombination are particularly critical.

Overall, this study highlights the strong potential of copper-based redox shuttles, when paired with appropriately designed organic dyes, as promising, efficient, and cost-effective alternatives for next-generation, high-performance DSSCs.

An interesting paper of Ganesan Shanmugam et al. combined the use of copper redox mediators with nanoparticles [24].

**Figure 8:**
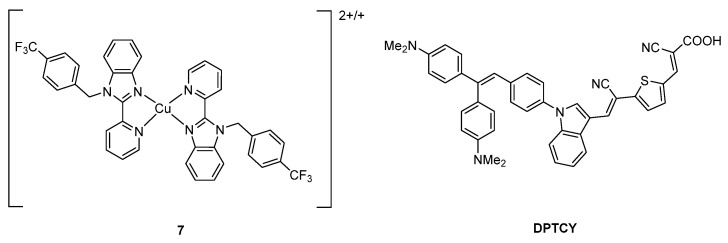
Molecular structure of Cu(I/II) redox couple **7** and of dye DPTCY [24].

The study focuses on improving the long-term stability and efficiency of dye-sensitized solar cells (DSSCs) through the development of a novel quasi-solid-state electrolyte (QSSE) that combines copper redox couples with biopolymer blends and metal oxide nanoparticles. In this work, ZnMoO_4_ nanoparticles were synthesized and compared with ZnO and MoO_3_ additives within a pectin–locust bean gum polymer matrix containing a Cu(I/II) redox system, [Cu(ptpbi)]^+^/^2+^ (ptbbi = [2-(pyridin-2-yl)-1-(4-(trifluoromethyl) benzyl)-1H-benzo[d]imidazole]), **7**, [24], along with an organic additive and DPTCY dye (see Figure 8).

Comprehensive material characterization and photovoltaic measurements revealed that the incorporation of metal oxide nanoparticles significantly enhances ionic conductivity, suppresses charge recombination, improves electron transport, and broadens light absorption. These effects are attributed to improved charge mobility within the quasi-solid matrix and better interfacial contact between the electrolyte and the photoanode.

Among the investigated systems, the ZnMoO_4_-based electrolyte exhibited superior performance. This behavior is mainly associated with its distinctive three-dimensional flower-like morphology, which promotes faster ion diffusion and more efficient interfacial charge transfer. As a result, the device shows enhanced photovoltaic performance along with improved operational stability.

This work demonstrates the effectiveness of combining copper-based redox mediators with structured nanomaterials and biopolymer electrolytes, providing a promising strategy for the development of stable and efficient quasi-solid-state DSSCs.

In another paper from Chen-Yu Yeh and Tzu-Chien Wei et al., they investigated how additives affect the degradation of DSSCs containing copper(II/I) complex redox couples [25]. So, this study investigates how different methylpyridine additives interact with the copper-based redox couple [Cu(dmp)_2_]^2+/+^, **8**, (dmp = bis(2,9-dimethyl-1,10-phenanthroline) in DSSCs, aiming to overcome the instability caused by the commonly used additive TBP (TBP = 4-tert-butylpyridine), which poisons the copper complex.

**Figure 9:**
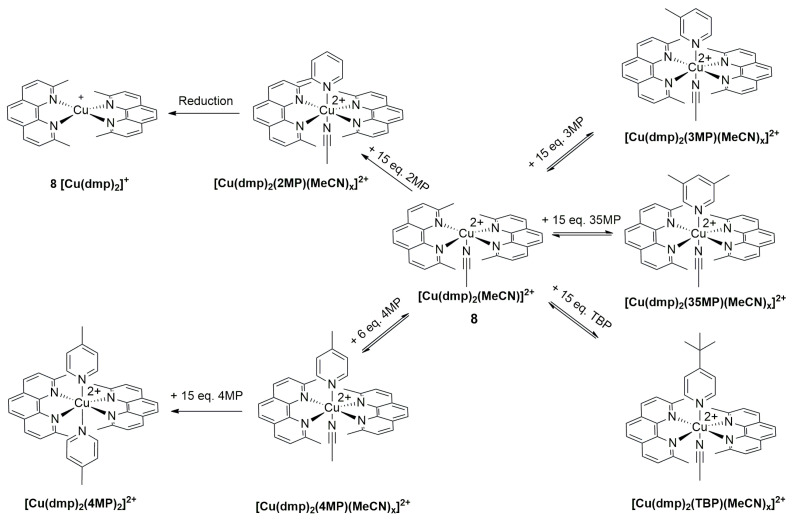
The reactions occurring from **8** inside the electrolyte system in the presence of different Lewis bases (x can be 1 or 0) [25].

The authors systematically examine four alternatives 2-methylpyridine (2MP), 3-methylpyridine (3MP), 4-methylpyridine (4MP), and 3,5-dimethylpyridine (35DMP) using spectroscopic, electrochemical, and device-level analyses, showing that the position and number of methyl groups strongly influence coordination behavior, redox properties, and charge transport (see Figure 9 to see the reactions occurring inside the electrolyte system in the presence of different Lewis bases). While 2MP reduces Cu(II) to Cu(I) and 4MP forms irreversible precipitates (both detrimental to performance), 3MP and 35DMP form stable, reversible complexes that maintain efficient charge transfer and reduce recombination.

As a result, DSSCs using 3MP and 35DMP achieve comparable or slightly better power conversion efficiencies than TBP while significantly improving long-term stability by mitigating steric hindrance and preserving electrochemical activity.

### 2.3. Miscellaneous

**Figure 10:**
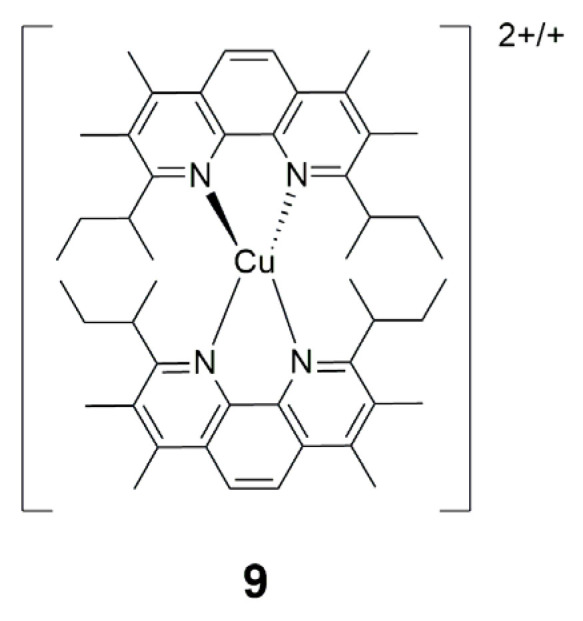
Molecular structure of the Cu(I/II) redox couple **9** [26].

A paper of Thomas W. Hamann et al. [26] introduces a novel dye-sensitized solar cell DSSC configuration called a “retro cell,” in which a single copper complex, [Cu(dsbtmp)_2_]^+^, **9**, (dsbtmp = bis(2,9-di(sec-butyl)-3,4,7,8-tetramethyl-1,10-phenanthroline, see Figure 10), simultaneously acts as both the light-absorbing chromophore and the redox shuttle, eliminating the need for separate dye and electrolyte components. The long excited-state lifetime of the copper complex enables it to diffuse through the electrolyte, inject electrons into TiO_2_, and then be regenerated at the counter electrode, simplifying device design and reducing energy losses. Experimental results confirm the concept is viable, although limited by inefficient charge separation, and show that adding TBP is crucial for improving performance by reducing recombination and modifying the coordination environment of the oxidized copper species. The role of TBP is highly system-dependent: while it can poison copper redox couples in conventional DSSCs, in this “retro cell” configuration it instead improves performance by tuning the coordination environment and reducing recombination. Overall, the study demonstrates a new, simplified pathway for DSSCs while identifying key limitations and mechanisms that must be addressed to improve efficiency. The key achievement is the first successful demonstration of a “retro cell” concept, proving that a single copper complex can function as both sensitizer and redox mediator in a DSSC, significantly simplifying the device architecture and opening a new design strategy for solar energy conversion.

In a recent study, Soman and Devi [27] explore how different TiO_2_ morphologies used as scattering layers influence light management and performance in dye-sensitized solar cells employing an organic dye (Y123) and a copper-based electrolyte. Three architectures—spherical particles (TSs), twisted fibers (TFs), and hierarchical nanoflowers (TNFs)—were synthesized and systematically compared in terms of optical properties, charge transport, and photovoltaic behavior. The results show that morphology strongly affects photon scattering, dye loading, and electron dynamics: while TNF provides high surface area and dye adsorption, and TF enables efficient electron transport, TS delivers the most effective light scattering across the visible spectrum, leading to enhanced photon confinement and photocurrent generation. Consequently, device efficiency is mainly governed by the balance between light harvesting and recombination losses rather than dye loading alone. Interestingly, under indoor lighting conditions, the influence of the scattering layer becomes negligible, highlighting the reduced role of photon scattering at low light intensity and providing new insights into morphology-dependent performance.

The main achievement is the demonstration that sphere-like TiO_2_ scattering layers significantly enhance DSSC performance, achieving 5.26% efficiency with a 23.7% improvement over devices without a scattering layer, and outperforming even commercial scattering materials due to superior photon management and reduced recombination [27].

The journey through copper complexes for DSSC ends with an inspiring review of Marina Freitag [28], “From Zombies to Smart Devices: The Evolution of Dye-Sensitized Solar Cells for IoT Applications”, highlighting the transformative role of copper compounds in advancing device performance and stability. This paper reviews the evolution of dye-sensitized solar cells (DSSCs) toward “zombie” devices, highlighting the central role of copper coordination complexes as advanced redox mediators and hole-transport materials for efficient indoor photovoltaics. Copper compounds such as [Cu(dmp)_2_]^2+/+^ (dmp = 2,9-dimethyl-1,10-phenanthroline) and [Cu(tmby)_2_]^2+/+^ (tmby = 4,4′,6,6′-tetramethyl-2,2′-bipyridine) exhibit high redox potentials, low reorganization energies, and fast charge-transfer kinetics, enabling high photovoltages, efficient dye regeneration, and reduced recombination losses. A key breakthrough is that these copper compounds can self-assemble into quasi-solid conductive networks upon solvent evaporation, forming “zombie” DSSCs that combine the advantages of liquid and solid-state systems. These materials establish mixed-valence conduction pathways within the TiO_2_ scaffold, providing high conductivity, enhanced stability, and elimination of leakage issues, ultimately allowing high-performance indoor photovoltaics and integration into self-powered IoT (IoT = Internet of Things) devices [28].

Complete data for the devices prepared with the copper compounds discussed in the present work are reported in Table 1.

## 3. Conclusions

In conclusion, the most recent advances clearly demonstrate that copper complexes have evolved from promising alternatives to ruthenium systems into key enablers of high-performance and sustainable DSSCs. Significant progress has been achieved in all device components: copper-based dyes now benefit from improved anchoring strategies and light-harvesting concepts, while copper redox mediators have reached impressive levels of efficiency, stability, and tunability through rational ligand design and additive engineering. Notably, the combination of optimized copper electrolytes with tailored organic or porphyrin dyes has enabled power conversion efficiencies exceeding 10%, narrowing the gap with state-of-the-art systems and confirming the technological relevance of these materials. At the same time, breakthroughs such as aqueous and quasi-solid-state electrolytes, additive-controlled coordination environments, and innovative device concepts (e.g., “retro cells” and zombie DSSCs) highlight the versatility of copper chemistry in addressing long-standing issues related to volatility, toxicity, and long-term stability.

From the perspective of technological transfer, long-term stability represents a critical requirement, often outweighing efficiency as a key performance metric. While Ru-based DSSCs have historically set the benchmark in terms of durability, recent advances clearly indicate that copper-based systems are becoming increasingly competitive in this respect. In particular, several studies discussed in this review report remarkable stability for copper-mediated devices, including systems operating for more than 1000 h at elevated temperature and others retaining a high percentage of their initial efficiency over extended periods of operation [19,20]. These results demonstrate that, when appropriately designed, copper complexes can provide robust and reliable performance. Moreover, the development of advanced electrolyte formulations, such as aqueous and quasi-solid-state systems, as well as innovative concepts like “zombie” DSSCs, has significantly mitigated common degradation pathways related to volatility, leakage, and corrosion. As a result, although further improvements are still required to achieve industrial-level lifetimes under all conditions, copper-based DSSCs currently represent some of the most promising candidates for combining sustainability, efficiency, and long-term operational stability in next-generation photovoltaic technologies.

Despite these achievements, challenges remain, particularly in further improving light absorption in copper dyes, minimizing recombination processes, and ensuring long-term operational durability under real-world conditions. Future research will likely focus on integrated molecular and device engineering approaches, combining theoretical predictions with experimental validation, to fully exploit the potential of copper complexes. Overall, the rapid progress reported in the 2024–2025 period strongly supports the view that copper-based systems represent a viable and increasingly competitive platform for the next generation of efficient, low-cost, and sustainable dye-sensitized solar cells, especially for indoor and energy harvesting applications.

## Figures and Tables

**Table 1:** Photovoltaic data of solar cells produced with copper-based compounds **1**–**8** ^a^.

Entry	Dye	Redox Couple	V_oc_ (mV)	J_sc_ (mA cm^−2^)	FF	η (%)	Ref.
1	**1**	I^−^/I_3_^− b^	493 ± 3	4.60 ± 0.12	67 ± 1	1.53 ± 0.03	[17]
2	**1**	I^−^/I_3_^− c^	468 ± 22	5.18 ± 0.28	68 ± 1	1.64 ± 0.00	[17]
3	**1**	I^−^/I_3_^− d^	604 ± 1	4.40 ± 0.10	71 ± 1	1.88 ± 0.05	[17]
4	**1**	I^−^/I_3_^− e^	404 ± 9	1.15 ± 0.13	60 ± 6	0.28 ± 0.07	[17]
5	C106	**2** ^f^	255 ± 8	0.10 ± 0.02	16	0.004	[19]
6	C106	**2** ^f^	660 ± 6	2.55 ± 0.05	58	0.98	[19]
7	C106	**2** ^f^	736 ± 5	2.49 ± 0.05	57	1.05	[19]
8	C106	**2** ^f^	736 ± 5	2.36 ± 0.05	53	0.92	[19]
9	C106	**2** ^f^	744 ± 5	2.05 ± 0.05	52	0.79	[19]
10	C106	**2** ^f^	732 ± 5	0.71 ± 0.03	71	0.37	[19]
11	C106	**2** ^g^	232 ± 7	0.08 ± 0.02	15	0.003	[19]
12	DA2	**3** ^h^	1112 ± 0.56	11.81 ± 0.56	76.1 ± 1.7	10.0 ± 0.2	[20]
13	FL1	**4·**TFSI ^i^	893 ± 10	12.1 ± 0.4	71.9 ± 0.9	7.78 ± 0.3	[21]
14	FL1	**4·**TFSI ^j^	895 ± 6	12.3 ± 0.5	74.8 ± 0.8	8.20 ± 0.2	[21]
15	FL1	**4·**PF_6_ ^j^	900 ± 10	12.7 ± 0.4	76.2 ± 0.6	8.80 ± 0.2	[21]
16	FL1 + XY1B	**4·**PF_6_ ^j^	940 ± 10	15.3 ± 0.1	75.2 ± 0.5	10.8 ± 0.1	[21]
17	SQ-HF	**5** ^k^	632	6.74	67	2.85	[22]
18	SQ-HF ^n^	**5** ^k^	690	10.83	69	5.15	[22]
19	DPTCY	**7** ^l^	905 ± 5	10.16 ± 0.13	65 ± 0.2	5.97 ± 0.06	[24]
20	DPTCY	**7** ^m^	845 ± 6	9.85 ± 0.05	61 ± 0.8	5.08 ± 0.12	[24]
21	DPTCY	**7** ^m^	860 ± 3	11.15 ± 0.08	56 ± 0.6	5.46 ± 0.11	[24]
22	DPTCY	**7** ^m^	880 ± 4	13.23 ± 0.10	53 ± 0.3	6.19 ± 0.14	[24]
23	Y123	**8** ^n^	962 ± 1.2	5.4 ± 0.3	58 ± 5	3.0 ± 0.2	[25]
24	Y123	**8** ^n^	1051 ± 0.2	11.7 ± 0.5	76 ± 1	9.3 ± 0.2	[25]
25	Y123	**8** ^n^	1062 ± 0.6	10.2 ± 0.3	73 ± 3	7.9 ± 0.2	[25]
26	Y123	**8** ^n^	1073 ± 0.3	12.0 ± 0.4	74 ± 3	9.6 ± 0.2	[25]
27	Y123	**8** ^n^	1066 ± 1.2	11.7 ± 0.1	73 ± 2	9.2 ± 0.4	[25]

^a^ Under AM 1.5 simulated light source, TiO_2_ employed as semiconductor; ^b^ LiI 100 mM + I_2_ 50 mM + 1,2-dimethyl-3-ethylimidazolium iodide 600 mM in ACN; ^c^ LiI 100 mM + I_2_ 50 mM + 1,2-dimethyl-3-ethylimidazolium iodide 600 mM + guanidinium thiocyanate + 100 mM in ACN; ^d^ as in note c but with the addition of TBP 0.5 M; ^e^ KI 4 M + I_2_ 20 mM in water saturated with CDCA (CDCA = chenodeoxycholic acid); ^f^ Cu(I) 0.1 M + Cu(II) 0.05 M + LiOAc 0.1 M + (HNMBI)OAc in water ((HNMBI)OAc = N-methylbenzimidazolium acetate); concentration of (HNMBI)OAc = 1.3 M in entry 6, 2.3 M in entry 7, 2.85 M in entry 8, 3.35 M in entry 9, 4.69 M in entry 10; ^g^ 3.35 M (EMImI)OAc ((EMImI)OAc = 1-Ethyl-3-methylimidazolium acetate) instead of (HNMBI)OAc; ^h^ Cu(I) 0.2 M + Cu(II) 0.04 M + LiTFSI 0.1 M + TBP 0.6 M in ACN; ^i^ Cu(I) 0.2 M + Cu(II) 0.05 M + LiTFSI 0.1 M + TBP 0.5 M in ACN; ^j^ Cu(I) 0.1 M + Cu(II) 0.025 M + LiX 0.1 M + TBP 0.5 M in ACN (X = TFSI in entry 14, X = PF_6_ in entries 15–16); ^k^ electrolyte composition not reported; ^n^ in the presence of CDCA; ^l^ electrolyte mixture obtained by addition of a stock solution of Cu(I) 0.2 M + Cu(II) 0.02 M + LiClO_4_ 0.1 M + MPP 0.1 M in ACN (MPP = 5-methyl-2-(pyridin-2-ylthio) pyridine) to a mixture of pectin and locust bean gum (1:1) in water and stirred at 50–60 °C; ^m^ as in note t but with the addition of ZnO in entry 20, of MoO_3_ in entry 21, of ZnMoO_4_ in entry 22; ^n^ Cu(I) 0.2 M + Cu(II) 0.04 M + LiTFSI 0.1 M + and LB 0.6 M in ACN (LB = Lewis Base): LB = 2-Me-py in entry 23, 3-Me-py in entry 24, 4-Me-py in entry 25, 3,5-diMe-py in entry 26, 4-*t*Bu-py in entry 27.

## Data Availability

We declare that all data used in the present paper are reported inside the manuscript itself.
